# Ultrasonic-Assisted Extraction of Antioxidants from *Perilla frutescens* Leaves Based on Tailor-Made Deep Eutectic Solvents: Optimization and Antioxidant Activity

**DOI:** 10.3390/molecules28227554

**Published:** 2023-11-12

**Authors:** Pengfei Jiao, Xingmei He, Shihua Ma, Shengping Wang, Qiuhong Niu

**Affiliations:** Research Center of Henan Provincial Agricultural Biomass Resource Engineering and Technology, College of Life Science and Agricultural Engineering, Nanyang Normal University, Nanyang 473061, China

**Keywords:** extraction, deep eutectic solvent, natural antioxidant, response surface optimization, Perilla leaf

## Abstract

The development of natural antioxidants to replace synthetic compounds is attractive. *Perilla frutescens* leaves were proven to be rich in antioxidants. The extraction of antioxidants from Perilla leaves via ultrasonic-assisted extraction (UAE) based on choline chloride-based deep eutectic solvents (DESs) was studied. Firstly, several DESs were prepared, and their extraction effects were compared. Secondly, the extraction process was optimized by single-factor experiments and response surface methodology (RSM). Finally, the optimization results were verified and compared with the results of traditional solvent-based UAE. The effects of solvents on the surface cell morphology of *Perilla frutescens* leaves were characterized by scanning electron microscopy (SEM). Choline chloride-acetic acid-based DES (ChCl-AcA) extract showed a relatively high ferric-reducing antioxidant activity (FRAP) and 2,2-diphenyl-1-picrylhyldrazyl radical scavenging rate (DPPH). Under the optimal operating conditions (temperature 41 °C, liquid–solid ratio 33:1, ultrasonic time 30 min, water content 25%, ultrasonic power 219 W), the experimental results are as follows: DPPH64.40% and FRAP0.40 mM Fe(II)SE/g DW. The experimental and predicted results were highly consistent with a low error (<3.38%). The values of the DPPH and FRAP were significantly higher than that for the water, ethanol, and butanol-based UAE. SEM analysis confirmed that ChCl-AcA enhanced the destruction of the cell wall, so that more antioxidants were released. This study provides an eco-friendly technology for the efficient extraction of antioxidants from *Perilla frutescens* leaves. The cytotoxicity and biodegradability of the extract will be further verified in a future work.

## 1. Introduction

*Perilla frutescens* is an annual herb in the Lamiaceae family. *Perilla frutescens* leaves are the most-used part of *Perilla frutescens*, which is used as a popular medicine, food, natural pigment, and spice in many countries such as China, India, Japan, and South Korea [1,2]. Modern pharmacological studies have shown that Perilla leaves contain rich bioactive ingredients, such as polyphenols, flavonoids, anthocyanins, etc., with activities including antioxidant, antitumor, and antibacterial [3,4]. Many nutritionists are working to develop natural antioxidants to replace synthetic compounds that are potentially dangerous for human health. The development of efficient and eco-friendly solvents is essential for the extraction of active compounds from medicinal plants and agricultural by-products.

Liquid–solid extraction is a common method for extracting antioxidants from plant and agricultural waste, usually using hot water or organic solvents as extractants. Although water is an eco-friendly solvent, it is very difficult to extract non-polar or water-insoluble compounds with water. Polyphenols and flavonoids with antioxidant activity mostly have low water solubility. Due to high toxicity, strong volatility, and flammability, organic solvents are not encouraged to be used in the food, cosmetic, and pharmaceutical industries. Therefore, there is a growing need to find greener alternatives to organic solvents. Ionic liquids and DESs are often used as green extraction solvents to replace traditional organic solvents. However, a DES is considered to be more environmentally friendly than ionic liquids, and easier to synthesize, and the cost is lower [5]. A DES is a low melting point mixture consisting of two or more natural components such as sugars, organic acids, and polyols to form strong hydrogen bonding complexes [6,7,8]. Due to low toxicity, easy biodegradation, low preparation cost, variety, and tunability, a DES has additional advantages in the selective extraction of target compounds. Therefore, a DES has been widely used in the separation and recovery of polyphenols, flavonoids, anthocyanins, and other active substances [9,10]. However, the high viscosity of DESs usually limits the mass transfer rate of the target compound in it, which leads to the slow extraction speed [11]. Therefore, some auxiliary means are needed to improve the extraction speed.

Modern extraction techniques including UAE [12], microwave-assisted extraction [13], high-pressure extraction [14], agitation-assisted extraction [15], and enzyme-assisted extraction [16] have received widespread attention for synergistically accelerating the extraction of compounds from medicinal plants or agricultural by-products. Among them, UAE can not only greatly reduce the consumption of solvents, labor, and energy, but also destroy the plant cell wall through cavitation, thereby improving the recovery of bioactive ingredients [17]. Many researchers have used UAE to extract antioxidants from *Perilla frutescens* [18,19,20]. Zhao et al. extracted polyphenols via combined UAE and cellulase-assisted extraction based on ethanol solution, and found that the extraction amount of polyphenols reached 28 mg GAE/g dry sample [18]. Yang et al. extracted caffeic acid and rosmarinic acid via supercritical CO_2_-based UAE, and found that ultrasound shortened the extraction time and reduced solvent consumption, and a high polyphenol yield could be obtained at a low extraction temperature [19]. Shang et al. used a DES-based microwave-assisted extraction technique to extract flavonoids from Perilla leaves and found that a choline chloride-malic acid-based DES achieved a significantly higher extraction yield than traditional solvents including methanol, ethanol, and water [21]. However, the application of a DES-based UAE to extract antioxidants from Perilla leaves has not been reported.

In this work, a DES-based UAE was used to extract antioxidants from Perilla leaves. Firstly, the extraction effects of different DESs were compared, and the better solvent was selected. With the temperature, liquid–solid ratio, ultrasonic power, ultrasonic time, and water content in the DES as the main process parameters, single-factor experiments and RSM based on the Box–Behnken design (BBD) were used to determine the interaction conditions and optimal conditions. Finally, SEM analysis of the Perilla leaves’ cell surface before and after extraction was carried out.

## 2. Results and Discussion

### 2.1. Screening of DESs

**Figure 1:**
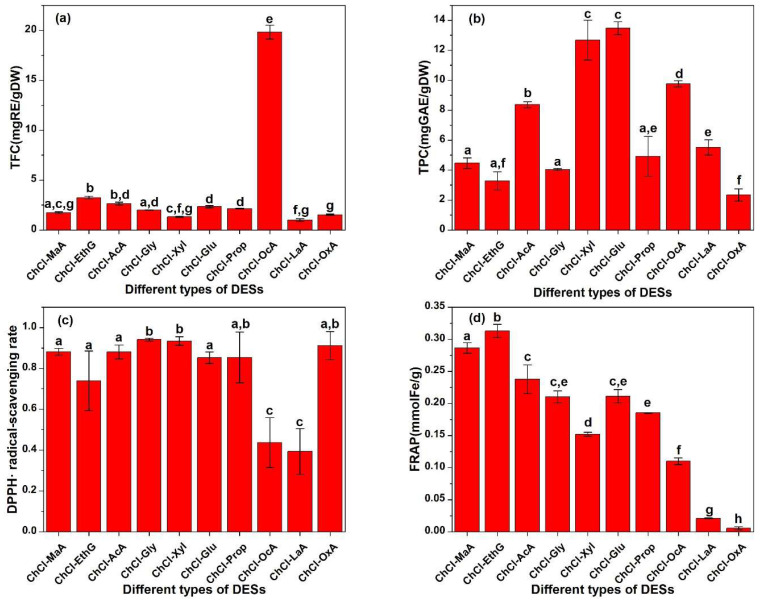
(**a**) TFC, (**b**) TPC, (**c**) DPPH, and (**d**) FRAP of different DES extracts. Different small letters on the top of bars represent significant statistical difference (*p* < 0.05).

The components of a DES determine its physicochemical properties, including polarity, viscosity, and solubility. The properties greatly affect the extraction yield of antioxidants. In order to screen effective solvents to extract more antioxidants from Perilla leaves, the TPC, TFC, DPPH, and FRAP of the extract obtained by different DESs are shown in Figure 1. In Figure 1c, the ChCl-MaA, ChCl-AcA, ChCl-Gly, ChCl-Xyl, and ChCl-OxA extracts exhibit a higher DPPH than other extracts. The ChCl-LaA extract shows the lowest DPPH, less than half that of the ChCl-AcA extract. In Figure 1d, the ChCl-MaA, ChCl-EthG, and ChCl-AcA extracts show a higher FRAP. The ChCl-OxA extract shows the lowest FRAP, which is 56.61 times lower than that of the ChCl-EthG extract. Considering both the DPPH and FRAP, the ChCl-MaA and ChCl-AcA extracts show excellent antioxidant activity. The DPPH of the two extracts is almost the same, but the FRAP of the ChCl-MaA extract is slightly higher than that of the ChCl-AcA extract. The results were similar to those obtained by Shang et al., who used microwave-assisted extraction to extract flavonoids from Perilla leaves. Shang et al. found that the content of flavonoids in the ChCl-MaA extract was higher than those in other DES extracts [21]. In the extraction of phenolic antioxidants from partridge leaf-tea, Wang et al. also found that the ChCl-MaA extract showed a higher antioxidant activity than other DES extracts [22]. By comparing the values of the TPC and TFC, it is found that the two indexes of the ChCl-MaA extract are lower than those of the ChCl-AcA extract. Different antioxidants differ in antioxidant capacity due to their different chemical structures. The types of antioxidants in the ChCl-MaA extract are different from those in the ChCl-AcA extract, and some polyphenols and flavonoids in the ChCl-AcA extract probably have low or no antioxidant activity. The fact that the antioxidant activity is not proportional to the concentration of polyphenols and flavonoids may also be due to the different mechanisms of antioxidant action of different substances, such as hydrogen atoms, single electron transfer, and metal chelation [23]. Faller et al. also reported the phenomenon that the antioxidant capacity is not proportional to the concentration of antioxidants [24]. The price of acetic acid ($700 per ton, data from https://m.cngold.org/price/jg8857758.html, accessed on 11 October 2023) is significantly lower than that of L-malic acid ($4237 per ton, data from https://www.chemicalbook.com/SupplyInfo_583754.htm, accessed on 11 October 2023). The density of acetic acid (1.1 ± 0.1 g/cm^3^, data from Chemspider database) was significantly lower than that of L-malic acid (1.6 ± 0.1 g/cm^3^). The lower solvent density is conducive to the solid–liquid separation after extraction. Therefore, ChCl-AcA was selected as the best extraction solvent for the subsequent experiment considering the extraction rate of antioxidants, cost saving, and convenience of operation.

The TFC of the ChCl-OcA extract was significantly higher than that of the other extracts. The possible reason is that the hydrophobicity of octanoic acid is significantly stronger than that of other solvents, and flavonoids usually have a strong hydrophobicity due to more benzene rings, so the hydrophobic interaction between octanoic acid and flavonoid molecules makes the extraction rate of flavonoids high. In Figure 1a, as for the TPC, the extraction rates of ChCl-Xyl and ChCl-Glu are higher than those of other solvents, possibly because there are more hydroxyl groups in the sugar molecules, which can form strong hydrogen bonds with the hydroxyl and carboxyl groups in polyphenol molecules, resulting in high extraction rates.

### 2.2. Single-Factor Experiments

**Figure 2:**
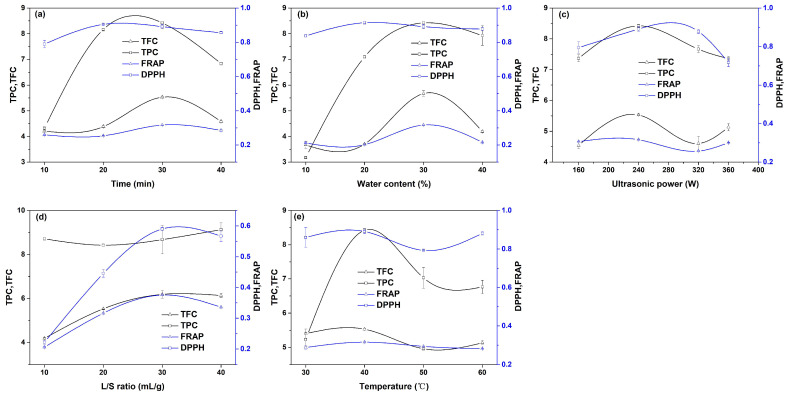
Single-factor experimental results: (**a**) ultrasonic time, (**b**) water content in DES, (**c**) ultrasonic power, (**d**) solid–liquid ratio, and (**e**) extraction temperature.

In order to evaluate the influence of single factors on the extraction effect and determine the appropriate range of variables, single-factor experiments were designed. The extraction experiments were carried out at different ultrasonic times (10, 20, 30, and 40 min) by fixing other operating conditions (ultrasonic power 240 W, temperature 40 °C, liquid–solid ratio 20:1, water content in DES 30%). The extraction results are shown in Figure 2a. In the Figure, the values of the TPC, TFC, and DPPH show the same change trend, increasing gradually with the extension of the ultrasound time, and then decreasing gradually. When the ultrasonic time is long, the reason for the decline in the above three values may be that the long-term ultrasonic treatment leads to the structural destruction of some antioxidants [25]. Huang et al. also found a similar phenomenon in the study on the extraction of crocins from gardenia fruit with a DES [12]. With the increase in the extraction time from 3 s to 1020 s, the yield of crocins showed a trend of first increasing and then decreasing. In our work, when the ultrasonic time is 20 min, the DPPH reaches the maximum value. The FRAP value hardly changes with the ultrasound time extending from 10 min to 20 min. The FRAP increases as the ultrasound time is extended, and reaches the maximum value at 30 min, and then begins to decline. Therefore, the extraction time range for response surface optimization was selected from 10 min to 40 min. By fixing other variables (ultrasonic power 240 W, temperature 40 °C, liquid–solid ratio 20:1, ultrasonic time 30 min), the extraction experiments were carried out under different water contents (10%, 20%, 30%, 40%), as shown in Figure 2b. The values of the TPC, TFC, FRAP, and DPPH all increase first and then decrease with the increasing water content. The reason for the increase in the four indexes may be that the increasing water content reduces the viscosity of a DES, which is conducive to the diffusion of antioxidants in the solvent. The reason why the four indexes decrease with the increasing water content may be that most of the antioxidants have a strong hydrophobicity and low solubility in water, resulting in a low extraction rate. The TPC, TFC, and FRAP reach their maximum value at 30% water content, while the DPPH reaches its maximum value at 20% water content. Therefore, the water content range in the DES for RSM was selected from 10% to 40%. Other variables were fixed (temperature 40 °C, liquid–solid ratio 20:1, ultrasonic time 30 min, water content 30%), and extraction experiments were carried out under different ultrasonic powers (160 W, 240 W, 320 W, 360 W), as shown in Figure 2c. In the range of 160 W to 320 W, the values of the TPC, TFC, FRAP, and DPPH all increase first and then decrease with the increasing power, and the maximum values are at 240 W. As the ultrasonic power continues to increase, the TPC and DPPH continue to decrease, but the TFC and FRAP show a slightly increasing phenomenon. According to the overall change trend of the four indicators, the ultrasonic power range for RSM was 160 W to 320 W. Other operating variables were kept unchanged (ultrasonic power 240 W, temperature 40 °C, ultrasonic time 30 min, water content 30%), and the liquid–solid ratio (10:1, 20:1, 30:1, 40:1) was changed to carry out the extraction experiments. The results are shown in Figure 2d. The values of the TFC, FRAP, and DPPH show the same change trend of first increasing and then decreasing with the increase in the liquid–solid ratio. At the liquid–solid ratio of 30:1, all three indexes reach the maximum value. The reason for the increase in the three indexes is that with the increase in the liquid–solid ratio, the concentration of antioxidants in the solvent increases slowly, which increases the mass transfer force of antioxidants in the Perilla leaf powder and causes more antioxidants to be released into the solvent. The reason for the drop in the three indicators may be that the solvent requires more energy when the liquid–solid ratio is too large, resulting in insufficient energy to break down the cell wall. Considering the overall trend, the liquid–solid ratio for RSM was selected from 20:1 to 40:1. Other operating variables were kept unchanged (ultrasonic power 240 W, ultrasonic time 30 min, liquid–solid ratio 20:1, water content 30%), and the solvent temperature (30 °C, 40 °C, 50 °C, 60 °C) was changed to carry out the extraction experiments, as shown in Figure 2e. In the range of 30 °C to 50 °C, with the increasing temperature, the four indexes showed a similar change trend of first increasing and then gradually decreasing, and reached the maximum value at 40 °C. The initial increase in the four indexes may be due to the increase in the temperature, which increases the diffusion and dissolution rate of molecules, reduces the viscosity of the solvent, and leads to more antioxidants being released into the solvent. The reason for the decline may be that high temperatures cause a change in the structure of some antioxidants. According to the overall change trend, the temperature range for RSM was selected from 30 °C to 50 °C.

### 2.3. Model Fitting and Response Surface Analysis

Several experiments based on BBD were carried out. The independent variable levels and the experimental data are represented in Table 1. The experimental values for the DPPH (32.43–65%) and FRAP (0.111–0.430 mM Fe(II)SE/g DW) indicated that the optimization of the UAE parameters was of great importance for obtaining high amounts of antioxidants. The relationship between the responses and variables is described as Equations (1) and (2):

(1) $$\mathrm{FRAP}=0.42+0.018A\;-\;0.025B\;-\;0.03C\;-\;0.032D\;-\;0.022E\;-\;0.062\mathrm{AB}\;-\;0.058\mathrm{AC}\;+0.024\mathrm{AD}\;-\;0.021\mathrm{AE}\;-\;0.032\mathrm{BC}\;-\;0.065\mathrm{BD}\;-\;0.036\mathrm{BE}\;-\;0.072\mathrm{CD}\;-\;0.087\mathrm{CE}\;+0.042\mathrm{DE}\;-\;0.06A^{2}-\;0.096B^{2}-\;0.064C^{2}-\;0.067D^{2}-\;0.09E^{2}$$

(2) $$\mathrm{DPPH}=61.66+2.92A+5.8B+1.64C+8.12D+2.23E+3.91\mathrm{AB}+3.34\mathrm{AC}\;-0.42\mathrm{AD}+0.05\mathrm{AE}\;-\;1.5\mathrm{BC}\;-\;2.21\mathrm{BD}\;-\;0.55\mathrm{BE}+0.76\mathrm{CD}\;-\;2.1\mathrm{CE}\;-\;3.16\mathrm{DE}\;-\;3.91A^{2}-\;8.44B^{2}-\;3.32C^{2}-\;4.68D^{2}-\;9.07E^{2}$$

ANOVA of the regression data was used to determine the statistical significance of the two quadratic polynomial models. The results for the ANOVA are shown in Table 2 and Table 3.

The experimental data of the FRAP and DPPH can be well predicted by the two models (*R*^2^ of the two parameters are 0.980 and 0.927, respectively, and the differences between the predicted *R*^2^ and adjusted *R*^2^ are less than 0.2). The CV values of the FRAP and DPPH were 5.52% and 6.11%, respectively, less than 10%, indicating a high reliability and accuracy of the experimental data. The Adeq Precision values for the FRAP and DPPH are 27.48 and 15.13, respectively, which are greater than 4 and indicate a desirable signal-to-noise ratio. A large F-value and a small *p*-value mean that the corresponding term has a large significance. As can be seen from Table 2 and Table 3, both regression models are statistically significant because the F-values for the models are large (60.68 and 15.78), and the *p*-values for the models are less than 0.0001. In the lack-of-fit analysis, the *p*-values of the FRAP and DPPH data fitting were 0.0812 and 0.0551, respectively, which were greater than 0.05, indicating that the models successfully established the functional relationship between the response values and variables.

**Figure 3:**
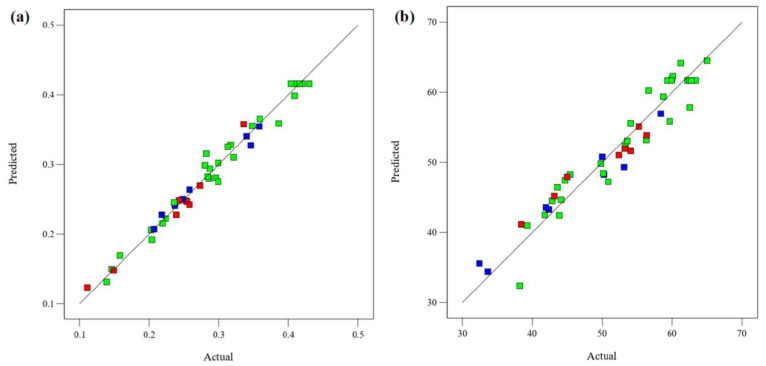
Predicted and experimental data for (**a**) FRAP and (**b**) DPPH.

**Figure 4:**
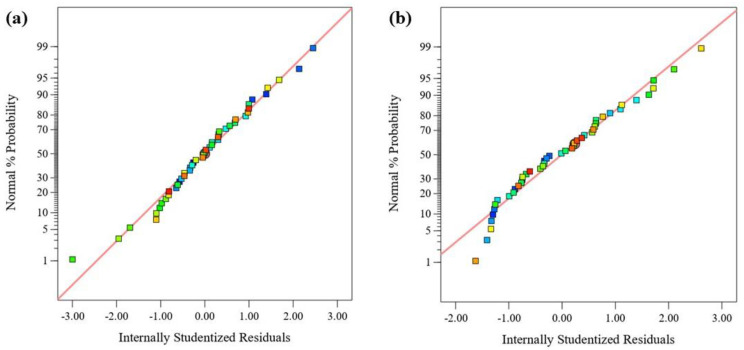
Normal probability for residuals for (**a**) FRAP and (**b**) DPPH.

The relationship between the experimental and the model’s predicted responses (FRAP and DPPH) is shown in Figure 3. Most of the data points in the figure closely fit the straight lines, indicating that the models can well explain the experimental results. The normal probability plot of the residuals is used to diagnose and prove errors from the assumptions and predictions of the model. The errors with the homogeneous variance normal distribution are not correlated with each other. The residual is defined as the difference between the observed and predicted values in a regression analysis. Figure 4 shows the normal probability diagram of the residuals of the models. There are negligible violations of the assumptions made. This satisfactory residual normal graph confirms the assumption of the models and the independence of the residuals.

If the *p*-value is less than 0.05, the item is statistically significant. In Table 2, the *p*-values of all items are less than 0.05, indicating that all items are statistically significant model items. Linear terms B, C, D, and E, quadratic terms A^2^, B^2^, C^2^, D^2^, E^2^, as well as interaction terms AB, AC, BD, CD, CE, and DE, have extremely significant effects on the FRAP (*p* < 0.0001). The effects of linear term A and the interaction terms AD, BC, and BE on the FRAP are highly significant (*p* < 0.01). By comparing the F-values, the order of factors affecting the FRAP is the liquid–solid ratio > power > water content > temperature > time. In Table 3, the *p*-values of A, B, C, D, E, AB, AC, A^2^, B^2^, C^2^, D^2^, and E^2^ are all less than 0.05, which are all statistically significant model items, while the other items are not statistically significant. The linear terms B and D and quadratic terms B^2^ and E^2^ have extremely significant effects on the DPPH (*p* < 0.0001). The effects of linear terms A and E and quadratic terms A^2^, C^2^, and D^2^ on the DPPH are highly significant (*p* < 0.01). By comparing the F-values, the sequence of factors affecting the DPPH is the liquid–solid ratio > water content > time > temperature > power.

**Figure 5:**
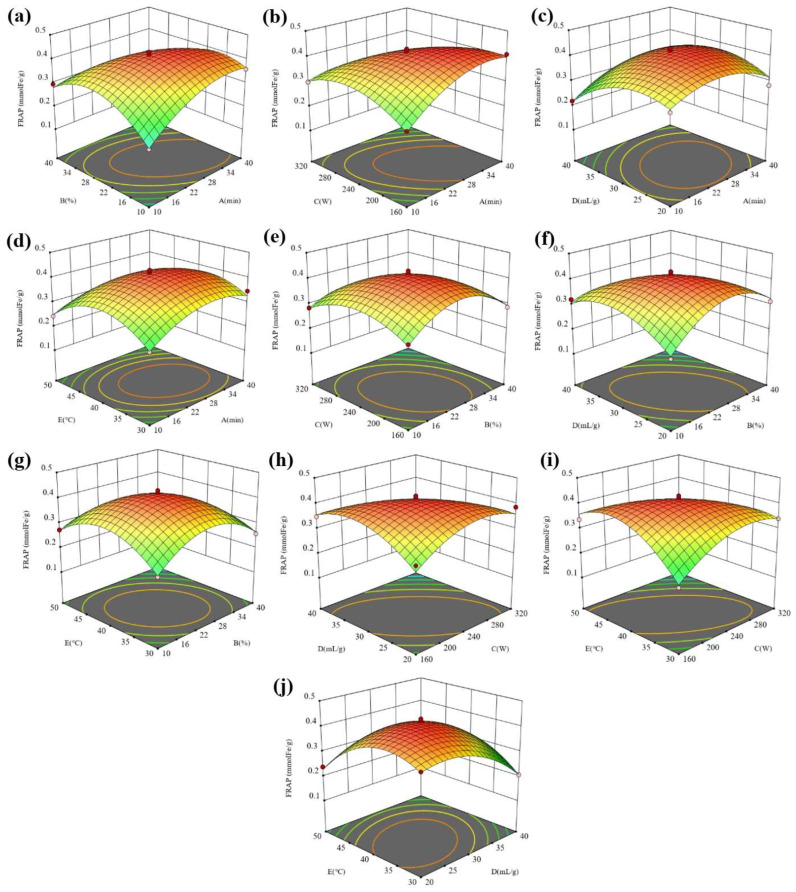
The 3D surface response maps of (**a**) AB, (**b**) AC, (**c**) AD, (**d**) AE, (**e**) BC, (**f**) BD, (**g**) BE, (**h**) CD, (**i**) CE, and (**j**) DE to FRAP.

Three-dimensional (3D) diagrams were used to analyze the effects of the variables and their interactions on the responses. The 3D response surface diagram is formed by using the established quadratic model. The two variables to be analyzed were distinguished in the selected experimental range to build a 3D surface plot with other variables held at the center level. Table 1 summarizes the range of variables identified in the single-factor experiments. The interaction effects of 10 variables, AB, AC, AD, AE, BC, BD, BE, CD, CE, and DE, are shown. The FRAP and DPPH reflect the antioxidant activity of the extract, and the higher the values, the higher the antioxidant activity. Figure 5 shows the interaction of five factors on the FRAP. The interaction of all factors on the FRAP is significant, indicating that the five factors have a good regulatory effect on the FRAP.

**Figure 6:**
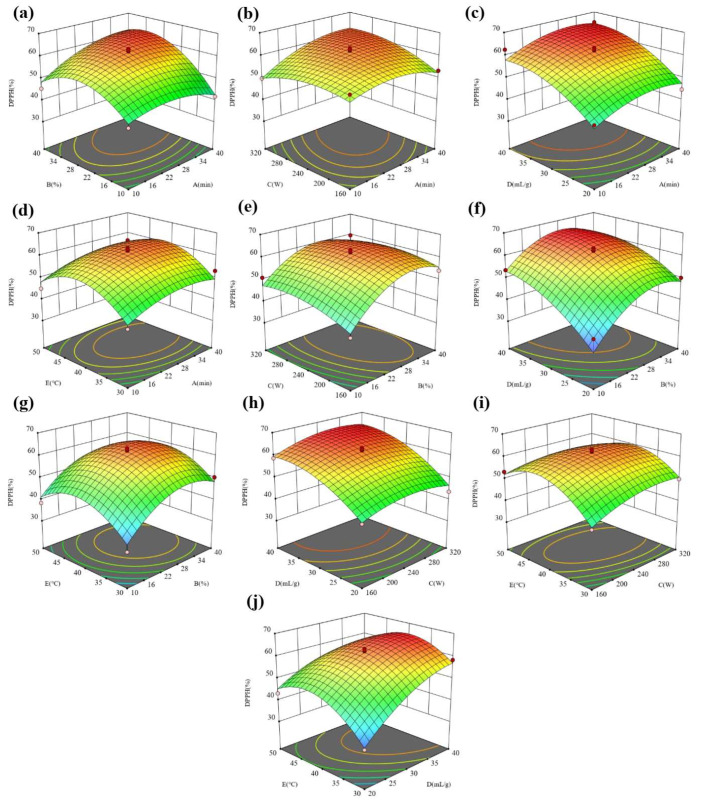
The 3D surface response maps of (**a**) AB, (**b**) AC, (**c**) AD, (**d**) AE, (**e**) BC, (**f**) BD, (**g**) BE, (**h**) CD, (**i**) CE, and (**j**) DE to DPPH.

In Figure 5a–d, under the given conditions of the water content, ultrasonic power, liquid–solid ratio, and temperature, the FRAP in the extract showed a trend of first increasing and then decreasing with the extension of the ultrasonic time. Especially for the water content and ultrasonic power, the increasing trend of the FRAP is more obvious with the extension of the ultrasonic time under the conditions of a low water content and ultrasonic power. However, at a high water content and ultrasonic power, the FRAP did not change significantly with the extension of the ultrasonic time. The difference in the variation trend is due to the interaction effect of the ultrasonic time and water content as well as the ultrasonic time and ultrasonic power. In Figure 5a,e–g, under the given ultrasonic time, ultrasonic power, liquid–solid ratio, and temperature conditions, the FRAP in the extract showed a trend of first increasing and then decreasing with the extension of the water content. Especially for the ultrasonic time and liquid–solid ratio, the increasing trend of the FRAP is more obvious with the increase in the water content at a low ultrasonic time and liquid–solid ratio. However, at a high ultrasonic time and liquid–solid ratio, the FRAP did not change significantly with the increasing water content. The difference in the variation trend is due to the interaction effect of the water content and ultrasonic time as well the water content and liquid–solid ratio. By comparing Figure 5b,e,h,i, it is found that the interaction between the ultrasonic power and time, ultrasonic power and liquid–solid ratio as well as the ultrasonic power and temperature have more significant effects on the FRAP than that between the water content and ultrasonic power. By comparing Figure 5c,f,h,j, it is found that the interaction between the liquid–solid ratio and water content, liquid–solid ratio and power as well liquid–solid ratio and temperature have more significant effects on the FRAP than that between the liquid–solid ratio and ultrasonic time. By comparing Figure 5d,g,i,j, it is found that the interaction between the temperature and power and temperature and liquid–solid ratio has a more significant impact on the FRAP than that between the temperature and other factors. Figure 6 shows the interaction effect of five factors on the DPPH. The interaction of the time and water content and time and power has a more significant influence on the DPPH than that of other factors.

### 2.4. Verification of Optimal Conditions for DES-Based UAE

To verify the reliability of the RSM optimization, a verification experiment was carried out under the optimal operating conditions predicted by the models. The predicted optimal operating conditions are as follows: temperature 41 °C, liquid–solid ratio 33:1, ultrasonic time 30 min, water content 25%, and ultrasonic power 219 W. The predicted response values are as follows: DPPH63.53% and FRAP0.414 mM Fe(II)SE/g DW. Under the optimal operating conditions, the experimental results are as follows: DPPH64.40% ± 1.09% and FRAP0.40 ± 4.35 × 10^−3^ mM Fe(II)SE/g DW. The experimental and predicted results are highly consistent with a low relative error value (<3.38%), indicating that the response surface analysis is suitable for the optimization of the operating conditions of the extraction process.

### 2.5. Comparison of Different Solvent-Based UAE

In Table 4, ChCl-AcA-based UAE showed the highest DPPH and FRAP. For the DPPH, the ChCl-AcA-based UAE was 28.04%, 77.98%, and 97.54% higher than that of the water-based UAE, ethanol-based UAE, and butanol-based UAE, respectively. For the FRAP, the ChCl-AcA-based UAE was 10.34%, 41.38%, and 51.72% higher than that of the water-based UAE, ethanol-based UAE, and butanol-based UAE, respectively. For the TPC, the ChCl-AcA-based UAE was lower than that of the water-based UAE, and higher than that of the ethanol-based UAE and butanol-based UAE. For the TFC, the ChCl-AcA-based UAE was lower than that of the water-based UAE, ethanol-based UAE, and butanol-based UAE. The possible reason is that some polyphenols and flavonoids in the water, ethanol, and butanol extract have low or no antioxidant activity. In conclusion, the ChCl-AcA-based UAE proposed in this paper does obtain more antioxidant active substances than that of other solvents.

### 2.6. SEM Analysis

**Figure 7:**
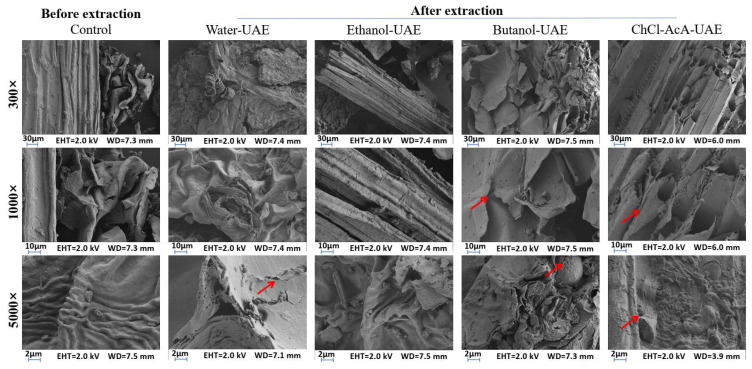
SEM analysis of Perilla leaves before and after extraction. Some cracks and holes can be seen where the red arrows indicate.

Figure 7 shows the effects of different solvents on the surface structure of Perilla leaf cells after UAE based on water, ethanol, butanol, and ChCl-AcA. Compared with freeze-dried Perilla leaves without solvent treatment, significant changes in the surface morphology of cells were observed. Before extraction, the surface of the leaves was relatively flat. After being treated with the above solvents, some holes and cracks appeared on the surface of the leaf cells. In particular, Perilla leaves treated with water and ChCl-AcA showed more holes and cracks. The ethanol-treated leaves showed no significant difference compared with the control group. Many researchers have reported that destroying cells can release more active compounds from the plant matrix [26,27]. ChCl-AcA can act as an effective solvent for breaking down the cell wall; thus, more antioxidants are released from the cells. This study is consistent with the results reported by de Almeida Pontes et al. that a DES can extract more phenolic compounds from olive leaves by damaging the cell structure [11].

## 3. Materials and Methods

### 3.1. Chemicals

Perilla leaves dried in the sun were purchased from Guangdong Fudonghai Pharmaceutical Co., Ltd. (Zhanjiang, China). The leaves were from the *Perilla frutescens* (L.) Britt. and they were picked between May and June 2023. In July 2023, they were transported from Guangdong to Henan Province under sealed conditions. Before use, the leaves were crushed by a pulverizer (MFJ-W317, Beijing Liren Technology Co., Ltd., Beijing, China) and passed through a 40-mesh screen to remove large particles. L-malic acid, n-octanoic acid, glycol, glycerin, 1,2-propylene glycol, and acetic acid were purchased from Shanghai Macklin Biochemical Co., Ltd. (Shanghai, China). Oxalic acid was bought from Shanghai Aladdin Biochemical Technology Co., Ltd. (Shanghai, China). Gallic acid, choline chloride, Folin–Ciocalteu reagent, sodium acetate, L-lactic acid, glucose, anhydrous aluminum chloride, 2,4,6-tripyridyl triazine, quinol dimethacrylate, D-xylose, and rutin trihydrate were obtained from Liaoning Cook Biotechnology Co., Ltd. (Fuxin, China). DPPH was acquired from Suqian Bozhiwei Trading Co., Ltd. (Suqian, China). These reagents are all analytical grade reagents and have not been further purified before use.

### 3.2. Preparation and Screening of DESs

A DES based on choline chloride was prepared via the method described by Wu et al. [28]. The hydrogen bond donors and acceptors were mixed in the molar ratios recommended by the relevant references shown in Table 5 [22,29]. Then, the solution was stirred in a thermostat water bath (Jintan district Baita new treasure instrument factory) at 80 °C until a uniform and stable liquid was formed, that is, a DES. An amount of 30% (*v*/*v*) of deionized water was added to reduce the viscosity of the DES. In order to preliminarily screen the best DES, 1 g Perilla leaf powder was added to a centrifuge tube containing 20 mL DES aqueous solution. Then, the centrifuge tube was placed in an ultrasonic cleaning machine (SB-5200DTD, Ningbo Scientz Biotechnology Co., Ltd., Ningbo, China) with a frequency of 40 kHz, pulsation cycle of 2.5 × 10^−5^ s, and extraction was carried out at 240 W and 40 °C for 30 min. The extract was obtained by filtering the mixture via qualitative filter paper (11 cm diameter) to remove solids. The contents of total polyphenols (TPC) and total flavonoids (TFC) as well as antioxidant activities of the extract were determined after a certain dilution.

### 3.3. Single-Factor Experiments

In order to select the optimal range of extraction conditions for RSM, single-factor experiments were carried out by changing one of the variables and fixing the other factors. The factors included ultrasonic power (160 W, 240 W, 320 W, 360 W), time (10 min, 20 min, 30 min, 40 min), liquid–solid ratio (10, 20, 30, 40), temperature (30 °C, 40 °C, 50 °C, 60 °C), and solvent water content (10%, 20%, 30%, 40%). The TPC, TFC, DPPH, and FRAP were the response variables of the single-factor experiments.

### 3.4. Box–Behnken Design

A five-factor three-level BBD model was used to select the optimal extraction conditions. The five factors were liquid–solid ratio, water content, ultrasonic power, ultrasonic time, and temperature. The DPPH and FRAP were selected as the responses of the optimization process due to the antioxidant activity not being completely positively correlated with the TPC and TFC found in this work. Forty-six experiments were designed by Design Expert 13 software, and the extraction experiments were carried out in random batches. Analysis of variance (ANOVA) was used as a diagnostic tool to determine the applicability of the proposed model. The coefficient of determination (*R*^2^, Adjusted *R*^2^, and Predicted *R*^2^) was used to evaluate the fitting quality of the model and measure the variability of the response variables.

### 3.5. Comparison of Different Solvent-Based UAE for Antioxidant Extraction

An amount of 1 gram of Perilla leaf powder was mixed with 37 mL of different solvents (Ch-Cl-AcA-based DES, deionized water, ethanol, and butanol) in a 50 mL centrifuge tube. Then, UAE was performed at 30 °C, liquid–solid ratio 30:1, and ultrasonic power 320 W for 40 min. The extract was obtained by filtering the mixture via qualitative filter paper (11 cm diameter) to remove solids. Then, the TPC, TFC, DPPH, and FRAP of the extract were determined after a certain dilution and compared.

## 4. Analytical Methods

### 4.1. Determination of Antioxidant Activity

The DPPH was determined using the method proposed by Lin et al. [30] with appropriate modification. The extract solution (50 μL) was mixed with 100 μmol/L of DPPH ethanol solution (400 μL) in a 2 mL centrifuge tube. The mixture was then left in a dark place at 25 °C for 20 min. Absorbance A_2_ was measured at 517 nm with ethanol as the blank control. The absorbance A_1_ was determined with anhydrous ethanol instead of DPPH solution. The absorbance A_0_ was determined using anhydrous ethanol instead of the sample solution. The DPPH was calculated using the following formula:

(3) $$\mathrm{DPPH}\left(\%\right)=\left(1-\frac{A_{2}-{\;A}_{1}}{A_{0}}\right)\;\times \;100\%$$

The method reported by Wang et al. [31] was used to determine the FRAP of the extract. Fresh FRAP reagents were prepared by mixing 0.3 mol/L acetic acid buffer (pH3.6), 20 mmol/L FeCl_3_·6H_2_O solution, and 10 mmol/L 2,4,6-tripyridinyl triazine solution at a 10:1:1 volume ratio. Then, 25 μL of diluted extract was mixed with 250 μL of FRAP reagent, and the absorbance was measured at 593 nm after the mixed solution was kept in a water bath at 25 °C for 30 min. With FeSO_4_ as the standard, the FRAP value was expressed as mmol Fe^2+^ equivalent per gram dry weight of the sample (mmol Fe(II)SE/g DW).

### 4.2. Determination of TPC

The TPC was determined by the colorimetric Folin–Ciocalteu method [5]. An amount of 1560 μL of deionized water, 40 μL of extract, and 100 μL of Folin–Ciocalteu reagent were added into a 15 mL polypropylene tube and shaken for 1 min. Then, 300 μL of freshly prepared 20% Na_2_CO_3_ solution was added to the mixture and shaken for 1 min. The mixture was stored in a dark place for 60 min, then the absorbance was measured at 750 nm using a UV–VIS spectrophotometer. Deionized water was used as a blank control. Gallic acid was the standard. The TPC was expressed as mg gallic acid equivalent per gram of perilla leaf (mg GAE/g).

### 4.3. Determination of TFC

The TFC was determined via the method proposed by Liu et al. [32]. A certain volume of extract was placed in a 25 mL volumetric flask. An amount of 5.0 mL of 0.1 mol/L aluminum trichloride solution and 2.5 mL of 0.2 mol/L acetate-sodium acetate buffer solution (pH5.2) were added. The solution was filled with 60% ethanol solution, heated in a water bath at 40 °C for 15 min, and then cooled to room temperature. The absorbance was measured at 405 nm with a color-developing agent as a blank control. Rutin was used as the standard substance. The TFC was expressed as mg rutin equivalent/g Perilla leaf (mg RE/g DW).

### 4.4. SEM Analysis

The Perilla leaf powder before and after extraction was freeze-dried and glued to the conductive adhesive, and the Quorum SC7620 sputtering coater was used to spray gold for 45 s and 10 mA. The sample morphology was then photographed using a ZEISS GeminiSEM300 scanning electron microscope with an accelerated voltage of 2 kV. The aperture size was 30 μm. The values of other determination parameters, such as the working distance and magnifications are shown in Figure 7.

## 5. Conclusions

An eco-friendly ChCl-AcA-based DES was prepared for the efficient extraction of antioxidants from Perilla leaves. The key operating parameters for enhancing the antioxidant yield were optimized using single-factor experiments and RSM. Under the optimal conditions of an ultrasonic temperature of 41 °C, liquid–solid ratio of 33:1, ultrasonic time of 30 min, water content of 25%, and ultrasonic power of 219 W, the experimental results were in good agreement with the predicted values. In addition, the ChCl-AcA-based UAE has a higher DPPH and FRAP than conventional solvent-based UAE. For the DPPH, the ChCl-AcA-based UAE was 28.04%, 77.98%, and 97.54% higher than that of the water-based UAE, ethanol-based UAE, and butanol-based UAE, respectively. For the FRAP, the ChCl-AcA-based UAE was 10.34%, 41.38%, and 51.72% higher than that of the water-based UAE, ethanol-based UAE, and butanol-based UAE, respectively. The SEM results showed that the ChCl-AcA-based DES could be used as an effective solvent to destroy the cell wall, thus allowing more antioxidants to be released. In summary, this work supports the potential use of a ChCl-AcA-based DES as a greener alternative to conventional organic solvents in the design of eco-friendly extraction methods for the recovery of phenolic compounds from Perilla leaves. Nevertheless, the non-toxicity and biodegradability of the DES and the extract still need to be further verified before their implementation in food, pharmaceutical, and cosmetic industries.

## Figures and Tables

**Table 1:** Experimental design and results.

Exp.	Variable					Response	
	Time	Water Content	Power	Liquid–Solid Ratio	Temperature	DPPH (%)	FRAP
1	25	25	240	40	30	58.36	0.207
2	40	25	240	20	40	44.66	0.282
3	25	25	160	30	50	53.21	0.336
4	25	10	160	30	40	39.26	0.286
5	10	25	160	30	40	56.31	0.253
6	25	25	320	40	40	61.23	0.146
7	25	25	240	20	50	43.15	0.239
8	25	25	240	40	50	55.23	0.254
9	10	25	240	40	40	62.51	0.219
10	40	40	240	30	40	59.97	0.204
11	25	10	240	20	40	38.21	0.236
12	10	25	240	30	50	44.97	0.243
13	25	40	240	30	30	50.26	0.258
14	10	40	240	30	40	45.39	0.296
15	10	10	240	30	40	42.84	0.203
16	40	25	240	30	30	53.12	0.346
17	25	25	320	30	50	52.39	0.111
18	40	25	240	30	50	56.36	0.258
19	25	25	240	30	40	63.34	0.415
20	25	40	240	20	40	50.12	0.313
21	25	25	160	30	30	42.38	0.218
22	10	25	240	30	30	41.93	0.248
23	25	40	240	40	40	56.63	0.139
24	40	25	320	30	40	60.12	0.224
25	25	40	240	30	50	54.05	0.149
26	25	25	240	20	30	33.65	0.358
27	40	25	160	30	40	53.29	0.409
28	25	25	320	30	30	49.98	0.340
29	25	40	160	30	40	54.05	0.287
30	25	25	240	30	40	59.31	0.409
31	10	25	320	30	40	49.78	0.299
32	25	25	240	30	40	59.92	0.404
33	25	10	240	30	30	32.43	0.237
34	25	10	240	30	50	38.43	0.273
35	25	25	160	40	40	58.72	0.348
36	25	40	320	30	40	59.65	0.158
37	10	25	240	20	40	43.86	0.317
38	25	25	160	20	40	44.13	0.299
39	40	25	240	40	40	65	0.280
40	25	25	320	20	40	43.59	0.386
41	25	25	240	30	40	62.72	0.430
42	25	25	240	30	40	62.19	0.420
43	25	25	240	30	40	62.46	0.416
44	40	10	240	30	40	41.77	0.359
45	25	10	240	40	40	53.56	0.321
46	25	10	320	30	40	50.85	0.284

**Table 2:** Results and parameters of ANOVA of response surface model for FRAP.

Source	Sum of Squares	Df	Mean Square	F-Value	*p*-Value
Model	0.30	20	1.51 × 10^−2^	60.68	<0.0001
A-Time	5.04 × 10^−3^	1	5.04 × 10^−3^	20.32	0.0001
B-Water content	9.75 × 10^−3^	1	9.75 × 10^−3^	39.31	<0.0001
C-Power	1.48 × 10^−2^	1	1.49 × 10^−2^	60.00	<0.0001
D-Liquid–solid ratio	1.66 × 10^−2^	1	1.66 × 10^−2^	67.09	<0.0001
E-Temperature	7.61 × 10^−3^	1	7.61 × 10^−3^	30.69	<0.0001
AB	1.54 × 10^−2^	1	1.54 × 10^−2^	61.99	<0.0001
AC	1.33 × 10^−2^	1	1.33 × 10^−2^	53.78	<0.0001
AD	2.30 × 10^−3^	1	2.30 × 10^−3^	9.29	0.0054
AE	1.72 × 10^−3^	1	1.72 × 10^−3^	6.94	0.0142
BC	4.03 × 10^−3^	1	4.03 × 10^−3^	16.26	0.0005
BD	1.68 × 10^−2^	1	1.68 × 10^−2^	67.61	<0.0001
BE	5.26 × 10^−3^	1	5.26 × 10^−3^	21.19	0.0001
CD	2.09 × 10^−2^	1	2.09 × 10^−2^	84.18	<0.0001
CE	3.01 × 10^−2^	1	0.03	121.35	<0.0001
DE	6.89 × 10^−3^	1	6.89 × 10^−3^	27.77	<0.0001
A^2^	3.05 × 10^−2^	1	3.05 × 10^−2^	123.08	<0.0001
B^2^	7.97 × 10^−2^	1	7.97 × 10^−2^	321.30	<0.0001
C^2^	3.55 × 10^−2^	1	3.55 × 10^−2^	143.27	<0.0001
D^2^	3.93 × 10^−2^	1	3.93 × 10^−2^	158.62	<0.0001
E^2^	7.00 × 10^−2^	1	0.07	282.22	<0.0001
Residual	6.20 × 10^−3^	25	2.48 × 10^−4^		
Lack of fit	5.80 × 10^−3^	20	2.89 × 10^−4^	3.57	0.0812
Pure error	4.05 × 10^−4^	5	8.11 × 10^−5^		
Cor total	0.31	45			

**Table 3:** Results and parameters of ANOVA of response surface model for DPPH.

Source	Sum of Squares	Df	Mean Square	F-Value	*p*-Value
Model	3113.87	20	155.69	15.78	<0.0001
A-Time	136.31	1	136.31	13.82	0.0010
B-Water content	537.89	1	537.89	54.52	<0.0001
C-Power	43.03	1	43.03	4.36	0.0471
D-Liquid–solid ratio	1054.14	1	1054.14	106.85	<0.0001
E-Temperature	79.57	1	79.57	8.07	0.0088
AB	61.23	1	61.23	6.21	0.0197
AC	44.62	1	44.62	4.52	0.0435
AD	0.7140	1	0.7140	0.0724	0.7901
AE	0.0100	1	0.0100	0.0010	0.9749
BC	8.97	1	8.97	0.9092	0.3494
BD	19.54	1	19.54	1.98	0.1717
BE	1.22	1	1.22	0.1238	0.7279
CD	2.33	1	2.33	0.2357	0.6315
CE	17.72	1	17.72	1.80	0.1922
DE	39.88	1	39.88	4.04	0.0553
A^2^	133.37	1	133.37	13.52	0.0011
B^2^	622.17	1	622.17	63.07	<0.0001
C^2^	96.24	1	96.24	9.76	0.0045
D^2^	191.28	1	191.28	19.39	0.0002
E^2^	718.61	1	718.61	72.84	<0.0001
Residual	246.63	25	9.87		
Lack of fit	233.22	20	11.66	4.35	0.0551
Pure error	13.42	5	2.68		
Cor total	3360.50	45			

**Table 4:** Comparison of UAE results based on different solvents.

	Water-Based UAE	Ethanol-Based UAE	Butanol-Based UAE	ChCl-AcA-Based UAE
TPC	10.94 ± 0.27	3.39 ± 0.03	2.76 ± 0.15	4.25 ± 0.05
TFC	2.80 ± 0.19	2.44 ± 0.28	2.21 ± 0.18	1.16 ± 0.05
DPPH	48.03% ± 1.4%	14.70% ± 4.68%	1.64% ± 1.15%	66.75% ± 1.56%
FRAP	0.26 ± 0.013	0.17 ± 6.70 × 10^−3^	0.14 ± 0.01	0.29 ± 0.015

**Table 5:** DESs prepared in this study.

No.	Solvent Abbreviation	Hydrogen Bond Acceptor	Hydrogen Bond Donor	Molar Ratio
1	ChCl-MaA	Choline chloride	Malic acid	1:1
2	ChCl-EthG		Ethylene glycol	1:2
3	ChCl-AcA		Acetic acid	1:2
4	ChCl-Gly		Glycerol	1:1
5	ChCl-Xyl		Xylose	2:1
6	ChCl-Glu		Glucose	1:1
7	ChCl-Prop		1,2-Propanediol	1:2
8	ChCl-OcA		Octanoic acid	1:2
9	ChCl-LaA		Lactic acid	1:2
10	ChCl-OxA		Oxalic acid	1:1

## Data Availability

Data are contained within the article.

## Abbreviations

AcA	Acetic acid
BBD	Box–Behnken design
ChCl	Choline chloride
DES	Deep eutectic solvent
DPPH	2,2-diphenyl-1-picrylhyldrazyl
EthG	Ethylene glycol
FRAP	Ferric-reducing antioxidant activity
Gly	Glycerol
Glu	Glucose
LaA	Lactic acid
MaA	Malic acid
OcA	Octanoic acid
OxA	Oxalic acid
Prop	1,2-Propanediol
RSM	Response surface methodology
SEM	Scanning electron microscopy
TPC	Content of total polyphenols
TFC	Content of total flavonoids
UAE	Ultrasonic-assisted extraction
Xyl	Xylose
